# The Christian Church and the Bodily Health
*A paper read at the Health Congress, held at Hastings under the presidency of Dr. B. W. Richardson


**Published:** 1889-10-19

**Authors:** F. Lawrence

**Affiliations:** Vicar of Weston, Yorkshire


### The Christian Church and the Bodily Health.*

By Rev. F. Lawrence, B.A., Vicar of Weston, Yorkshire.

The Mosaic Church gave much attention to the bodily health. The laws regulating isolation in cases of infectious diseases, such as leprosy, were the very perfection of quarantine; while the disposal of decaying substances in the earth has been acknowledged, in these enlightened days, to be the most salutary mode known.

The Christian Church has also, like her Founder, paid great regard to the health of the body. Recognising the precariousness of infant life she sanctions baptism at the house when there is sufficient cause. She bids all to keep the body in temperance, soberness, and chastity, and not to covet nor desire other men's goods, but to learn and labour truly to get their own living; thus supplying a perfect rule for keeping a sound mind in a sound body. She takes cognisance of the youth and maiden arrived at years of discretion, and requires them to renew their baptismal vows, to renounce the devil and all his works, the pomps and vanity of this wicked world, and all the sinful lusts of the flesh. To those who have not the gift of continency she recommends marriage as honourable for the mutual society, help, and comfort that the one ought to have of the other in sickness and in health. She rejoices with the mother after the hour of peril in child-birth, exhorts all to arrange their worldly affairs while in health, and, when sickness comes, strive to comfort and cheer. In extreme cases she allows the minister to communicate alone with the sick person. When death closes this earthly scene she mourns with the bereaved, and holds out the hope of a joyous resurrection. The corpse is to be borne either into the church or toward the grave, thus providing against the possible spread of infection; the body is to be made ready to be laid into the earth; and earth is to be cast upon it by some standing by, that is, upon the corpse or body, not the coffin. And such burial, when honestly carried out, is found to be so completely in accordance with natural law, that insects, birds, and beasts, wild and tame, practise the same. Moreover, the rubric directing such simple burial is not only the law of the Church, but also the law of sanitary science, and is consistent both with the welfare of the living, and due respect to the dead; and that it is feasible is shown in some of our old churchyards where, the burials having been few and simple, the ground has received the dead for centuries with harm to none.

Thus the Church is the great national sanitary agency, and her officers who have unique authority and influence in every parish in the land, are called upon to take an active part in promoting the bodily welfare of all, and among other things to endeavour to bring back the nation to the Church's sanitary "earth-to-earth" mode of burial.

* A paper read at the Health Congress, held at Hastings under the presidency of Dr. B. W. Richardson

A large proportion of the "insect powders" sold are made from the flowers of the pyrethrum, a plant which flourishes in the Caucasus. The only part of Europe where it grows is Dalmatia, and when the crop there is small the price of the Caucasian flowers becomes nearly double.

